# Introducing blood flow in kidney explants by engraftment onto the chick chorioallantoic membrane is not sufficient to induce arterial smooth muscle cell development

**DOI:** 10.1242/bio.059459

**Published:** 2022-07-06

**Authors:** Julia Tarnick, Jamie A. Davies

**Affiliations:** Deanery of Biomedical Sciences, University of Edinburgh, Edinburgh EH8 9XD, UK

**Keywords:** Kidney development, Arterial differentiation, CAM grafting

## Abstract

Kidney explant cultures are an important tool to gain insights into developmental processes, insights that can be used to develop strategies for engineering kidneys from stem cells. However, explants are not connected to a perfused vascular system. This limits their survival and limits physiological studies, for example of blood filtration, the main function of the kidney. Previous studies have shown that grafting kidneys onto avian chorioallantoic membrane (CAM) can establish perfusion and enable glomerular vascularization, but the realism and maturity of the resultant vasculature has not been examined. Here, we show that vasculature of kidney explants grafted onto CAM is very different from natural kidney vasculature, showing excessive growth of endothelial cells, absence of a hierarchical arterio-venous network and no vascular smooth muscle cell recruitment. The model therefore has serious limits.

## Abstract (precis)

**Summary:** The chorioallantoic membrane assay has been previously used to verify the vascularization potential of engineered organoids. We identified severe limitations of this assay, such as a lack of arterial maturity.

## INTRODUCTION

Studying organ growth *ex vivo* is a powerful tool for developmental and regenerative research as it allows observation of development as it happens, and the application of a range of experimental techniques that are not possible, for either practical or ethico-legal reasons, *in vivo*. However, cultured organ explants are not connected to any circulatory system, and this lack of circulation can impact their survival and function (Daniel and Cleaver, 2019; Vargas-Valderrama et al., 2020; Yu, 2021).

The kidney represents a valuable model system to characterize vascular development because renal blood vessels, nephrons and collecting ducts need to be in a precise spatial relationship to enable the concentration of urine and prevention of excessive fluid loss (Dantzler et al., 2011). Without this arrangement, kidneys cannot fulfil their primary physiological function. Additionally, it has been shown that while kidney explants, and renal organoids, contain endothelial cells, the maturation of these is severely limited under traditional culture methods, which is evidenced by the lack of vascularized glomeruli and arterial smooth muscle cell recruitment (Bernstein et al., 1981; Munro et al., 2017; Sariola et al., 1983). An effective filtration depends on the regulation of blood flow, which is in part provided through the myogenic response of the renal arteries (Burke et al., 2014), the absence or misplacement thereof is therefore likely to affect renal function. The lack of vascular maturity is often attributed to the lack of blood flow (Homan et al., 2019; Ryan et al., 2021). Consequently, connecting renal explants to the circulatory system of a host, thereby introducing flow through the renal capillary plexus, should promote vascular maturation.

One method of connecting renal explants to the circulation of a host is engraftment onto the chick egg chorioallantoic membrane (CAM), which allows vessel development to be observed relatively easily, as reported previously (Loughna et al., 1997; Preminger et al., 1980; Sariola et al., 1983). These studies reported development of vascularized glomeruli, which would be essential for renal filtration, and therefore represented an important improvement compared to traditional culture methods. Other aspects of vascular maturity, such as the recruitment of vascular smooth muscle cells, have, to the best of our knowledge, not yet been evaluated. Additionally, little has been reported on the overall anatomical realism and maturity of the developing vasculature. Having screened existing literature, we found a mention of the presence of arteries in CAM-engrafted chick mesonephroi (temporary kidneys) (Navarro et al., 2003), but did not come across a record where the presence of smooth muscle cell markers was tested in grafted murine metanephroi (permanent kidneys).

Given the apparent promise of the chick CAM system, based on the reports cited above, we have performed a careful study on the features and limitations of CAM culture in making realistic renal vasculature in explanted renal primordia. We find the method to have serious limitations: the engrafted explants contained an excess of capillaries, did not display a realistic arrangement of blood vessels, and the vessels that did form lacked maturity as indicated by their failure to recruit arterial smooth muscle cells.

## RESULTS AND DISCUSSION

### Embryonic kidneys graft onto the CAM with low efficiency, which is not enhanced by stimulation of VEGF expression

**Fig. 1.**
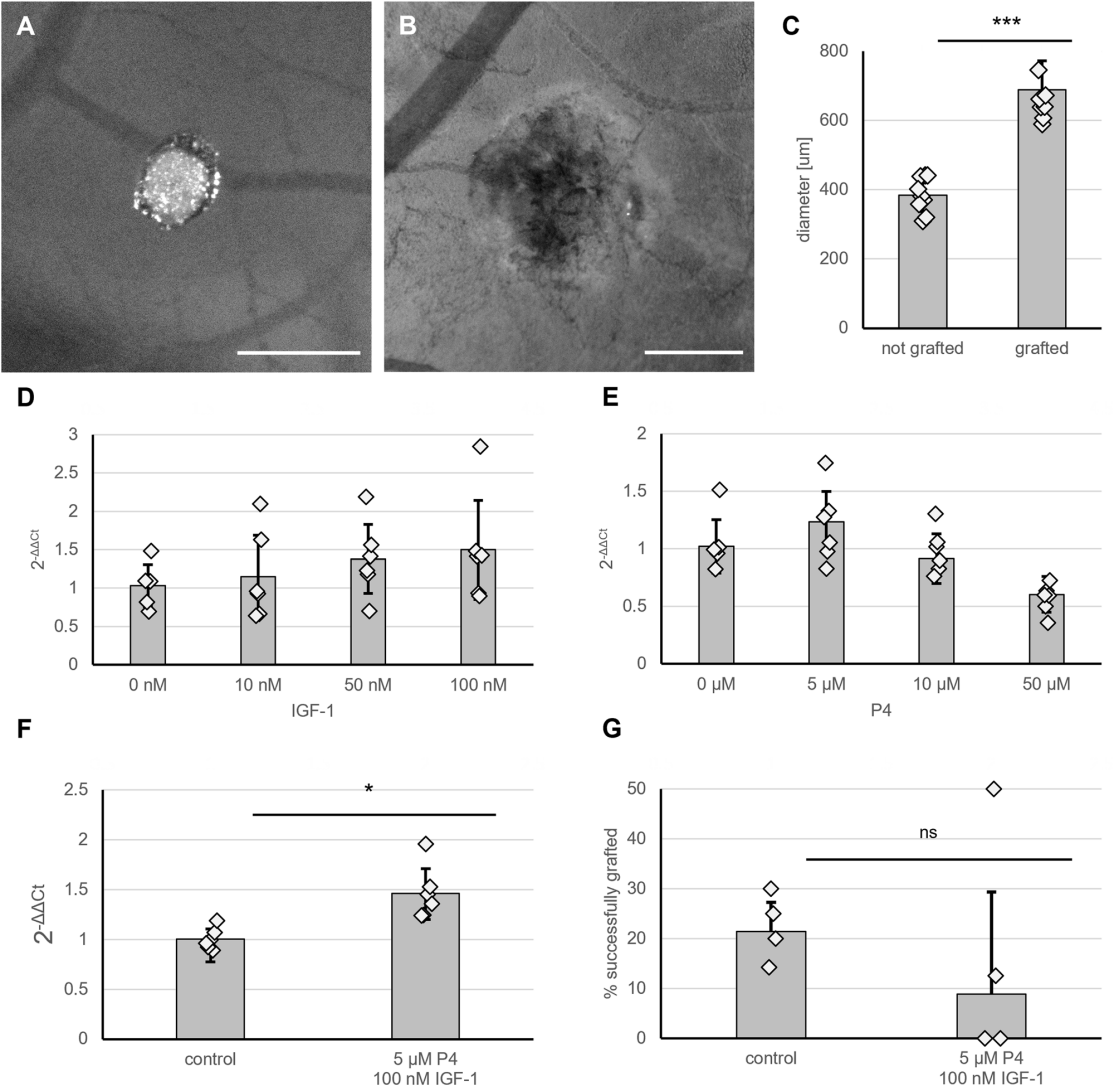
**Embryonic kidney explants (E11.5) graft with low efficiency.** (A) Kidney explants that appeared as a white disc were considered ungrafted. Scale bar: 500 µm. (B) Successfully grafted kidneys display visible size increase and blood vessels entering the kidneys. Scale bar: 500 µm. (C) Bar chart and scatter plot displaying difference in diameter between successfully grafted and ungrafted kidneys. Error bars display standard deviation. ***P<0.001 according to Student's *t*-test. (D) Induction of *Vegf* expression does not increase grafting efficiency. (E) Vegf expression after treatment with indicated doses of progesterone (P4), *N*=6. (F) Vegf expression after treatment with IGF-1, *N*=6. Treatment of kidney explants with P4 and IGF-1 increased Vegf expression by 50%. *P<0.05, Student's *t*-test, *N*=6. (G) Treatment with P4 and IGF-1 did not increase the grafting efficiency. ns, not significant. Scatter plot shows individual data points. The bar chart displays the average grafting efficiency across four experiments. During each experiment, four to 16 kidneys were grafted per treatment group. Error bars display standard deviation. Calculation of the 95% confidence interval indicated no statistical significance.

To allow vascularization by a host circulatory system, 3 day-cultured embryonic kidneys [embryonic day (E)11.5] were placed onto the CAM of day 7 chick embryos. After 3 days of culture *in ovo*, most explants appeared as shrivelled white discs (Fig. 1A), but between a quarter and a fifth of explants appeared healthy and increased in size (Fig. 1B and C). These contained multiple blood vessels that entered the kidney from the CAM, the sites of entry being apparently random with respect to the orientation of the grafted kidney (Fig. 1B). Kidneys displaying these characteristics were considered to be successfully grafted, while explants that appeared as shrivelled white discs were considered unsuccessful. Across four independent experiments, an average success rate of 22% was reached, the efficiencies in individual experiments varying between 14% and 30%. Due to varying survival rates of the chick embryos, the number of grafts obtained at the end point of the experiments varied between four and 16 per treatment group (Table S3).

*In vivo*, tissues secrete VEGF to attract blood vessels (Eremina et al., 2003; Neufeld et al., 1999); therefore, a higher expression of *Vegf* by the grafts might potentially increase grafting efficiency, as seen previously where kidneys soaked in VEGF before grafting connected more efficiently to the vasculature of a rat host (Xinaris et al., 2012). In kidneys, VEGF is secreted by the collecting ducts (Simon et al., 1995) and podocytes (Eremina et al., 2003). In order to stimulate the expression of VGEF within the collecting ducts, the cultured kidney explants were treated with the known *Vegf*-inducers progesterone (P4) (Sone et al., 1996) and insulin-like growth factor 1 (IGF-1) (Punglia et al., 1997), the receptors of which are expressed specifically in the collecting duct compartment (Harding et al., 2011; McMahon et al., 2008). To verify upregulation of *Vegf* transcription by these factors in kidneys, 48 h-cultured E11.5 kidney explants were treated with P4 and IGF-1 and cultured for additional 24 h, after which RNA was isolated for real-time RT-PCR analysis.

When used individually, neither P4 nor IGF-1 resulted in a statistically significant change in *Vegf* expression (Fig. 1D,E). A combination of 5 µM P4 and 100 nM IGF-1 increased the expression of *Vegf* by 50% (*P*<0.05) (Fig. 1F). However, this treatment with IGF-1 and P4 had no statistically significant effect on the grafting success rate as implied by calculation of the 95% confidence interval (CI) for the difference in proportions of grafted and ungrafted kidneys between treated and control samples (−0.03: 0.28) (Fig. 1G). While we cannot rule out that further increase of the *Vegf* expression could potentially increase the grafting efficiency, it does seems unlikely as treated explants showed a tendency to lower, albeit not statistically significantly lower, grafting success.

Most previous studies have not reported the efficiency of the grafting process. However, those which have reported a success rate between 40% using chicken egg CAM (Preminger et al., 1980) and 96% using quail egg CAM (Sariola et al., 1983). This is higher than we achieved. In the study conducted by Preminger et al. (Preminger et al., 1980), the grafting efficiency was increased by 23% through gentle abrasion of the CAM, suggesting that optimization of the grafting method, for example by using a harsher abrasion technique, might have a higher effect than pharmacological treatment of the grafts.

### Grafting of kidney explants onto the CAM increases the number of vascularized Bowman's capsules

**Fig. 2.**
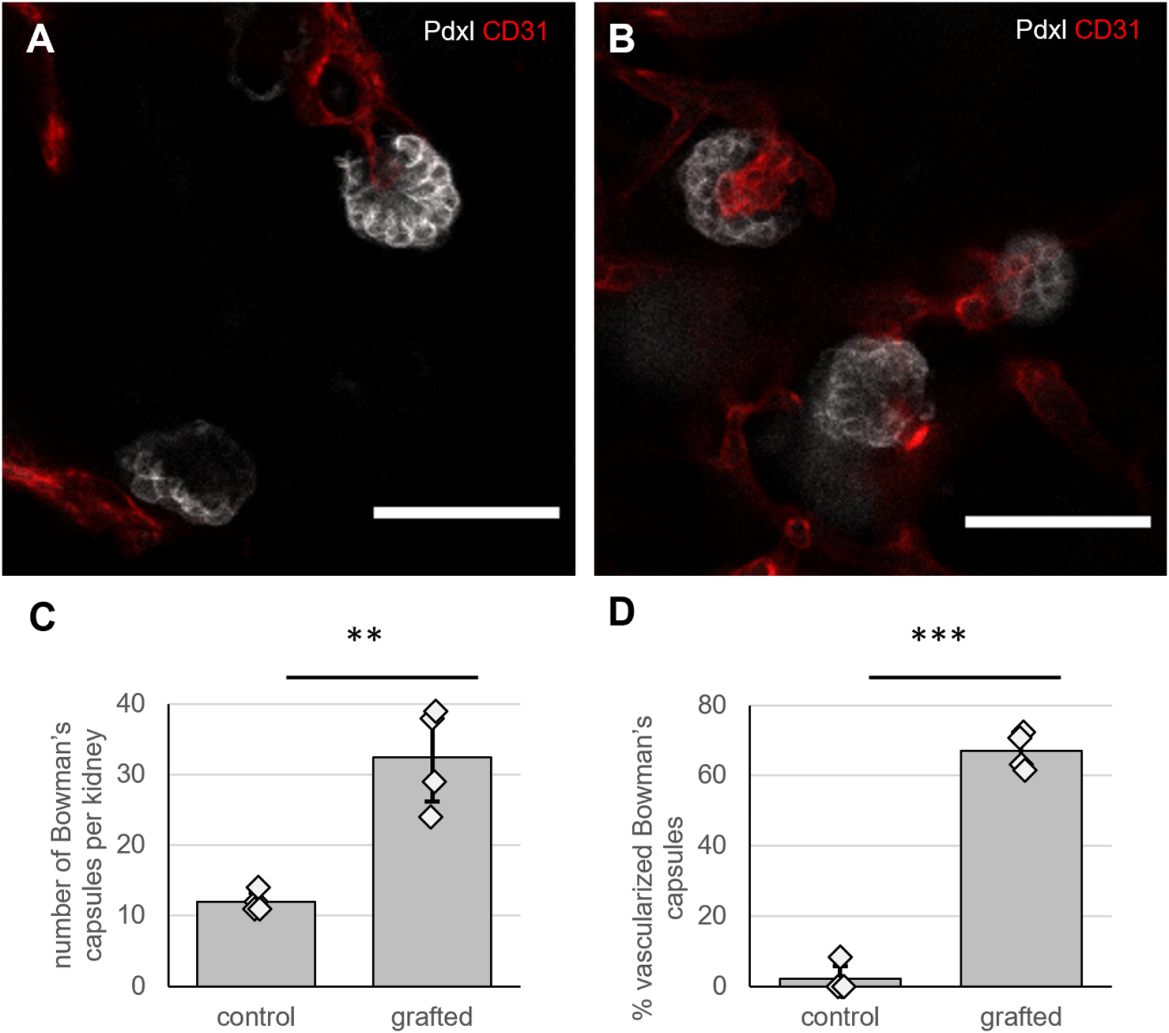
**Grafted kidneys contain more glomeruli and a higher percentage of vascularized glomeruli.** (A) Staining of kidneys cultured in collagen for 6 days for podocalyxin (Pdxl) and CD31 (red). One of the glomeruli appeared vascularized. Scale bar: 50 µm. (B) Staining of grafted kidneys after 3 days of *in ovo* culture for podocalyxin (Pdxl; white) and CD31 (red). Scale bar: 50 µm. (C) Total number of glomeruli found in grafted and control kidneys. *N*=4, error bars display standard deviation. **P<0.01 according to Student's *t*-test. (D) Percentage of vascularized glomeruli, *N*=4, error bars display standard deviation. ***P<0.001 according to two-proportion *Z*-test.

Consistent with previous studies (Garreta et al., 2019; Sariola et al., 1983), engraftment of kidney explants onto the CAM enhanced vascularization of the Bowman's capsules (Fig. 2A-D). Control kidneys grown in type I collagen for 6 days contained significantly fewer total Bowman's capsules (*P*<0.01, Student's *t*-test), and a glomerulus (Fig. 2A) was detected in only one of four samples. In contrast, grafted kidneys contained three times the number of Bowman's capsules (Fig. 2B). As the CD31 antibody used to stain endothelial cells did not detect the galline vessels within the CAM (Fig. S1), we could not determine the number of Bowman's capsules vascularized by host-derived blood endothelial cells. However, we noted that about 65% of the Bowman's capsules were vascularized by murine-origin blood vessels (Fig. 2B,C). The relatively large proportion of kidney-derived glomerular capillaries was unexpected, as previous studies displayed host-derived blood vessels as the primary origin of vascularized glomeruli in grafted kidneys (Ekblom et al., 1982; Sariola et al., 1984). The difference could be explained by using slightly older explants in this study (E11.5 compared to E11) and the process of preculturing the explants in collagen, which would allow endothelial cells present in the isolated explant to proliferate prior to grafting and therefore would lead to an increased proportion of already present capillaries in the kidney explants. It also has to be noted that our method would not detect glomeruli from host-derived blood vessels. We can therefore not estimate the proportion of glomeruli consisting solely of galline endothelial cells nor the proportion of hybrid glomeruli containing vessels of both species.

### The connection of kidney explants to the blood flow of the host is not sufficient to drive organotypic arterial maturation

While cultured kidneys and kidney organoids do contain endothelial cells, these typically do not differentiate into arteries and veins, meaning that an organotypic vascular network never forms (Munro et al., 2017; Murakami et al., 2019). The lack of blood flow is assumed to be a major reason for the limited maturity of blood vessels in culture (Homan et al., 2019; Ryan et al., 2021). Therefore, we aimed to determine whether the flow provided by the host embryo in the CAM system was sufficient to induce arterial smooth muscle cell recruitment within the graft, as seen in the host CAM vasculature. We observed flow in the vessels surrounding the grafts (Movie 1). To verify that blood was flowing in the vessels that entered the grafted kidney, we initially injected one of the CAM vessels with fluorescein-conjugated dextran. While this was suitable to visualize the flow in living grafts (Movie 2), we were unable to validate whether the flow would reach the capillaries inside the graft, as dextran, being sugar-based, cannot be crosslinked using standard fixatives such as paraformaldehyde (PFA). In order to be able to fix the injected dye for subsequent immunofluorescent staining, we opted to use AlexaFluor 594-conjugated hydrazide or an AlexaFluor 555-conjugated donkey-anti-mouse secondary antibody (DAM555).

**Fig. 3.**
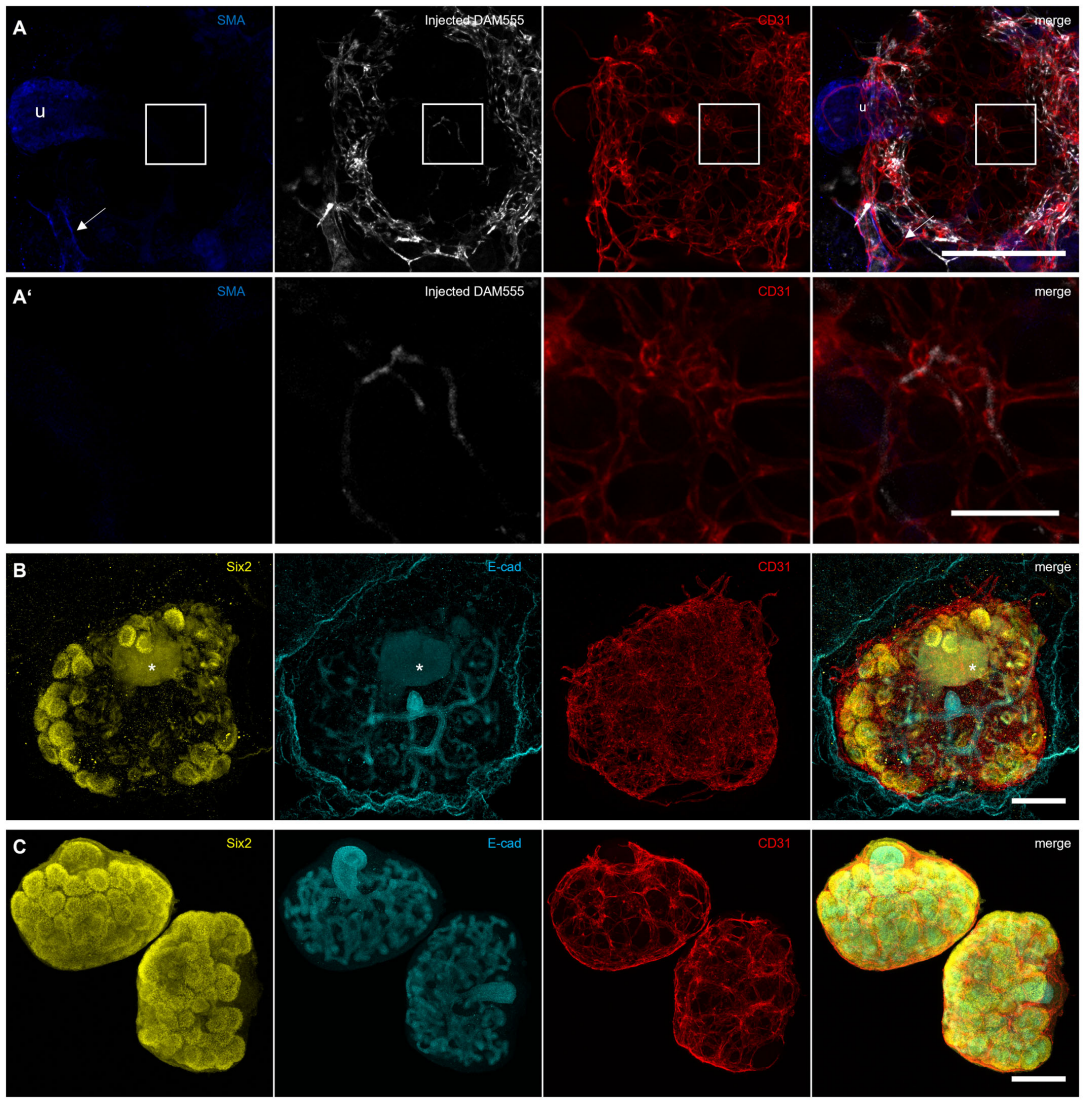
**Flow provided from the host is not sufficient to induce smooth muscle cell recruitment during arterial maturation.** (A) After injection of a donkey anti-mouse AlexaFluor555 antibody (DAM555) into the host vasculature, the graft was isolated and stained for smooth muscle actin (SMA) and CD31. SMA is visible around the ureter of the kidney (u) and in an artery of the host (arrow). Scale bar: 200 µm. (A′) Magnified area of A showing the injected DAM in the central capillaries of the graft. No smooth muscle actin-positive cells can be detected around the blood vessels. Scale bar: 50 µm. (B) Staining of the grafts for Six2, E-cadherin and CD31 reveals the presence of excess vasculature around the kidney. One of the three grafts analysed further displayed an abnormal cell mass (marked with *). Scale bar: 200 µm. (C) Kidney explants cultured in type 1 collagen for 6 days prior to staining with Six2, E-cadherin and CD31. Scale bar: 200 µm.

After, fixation and staining, several grafts had lost the signal of the injected dye, which had been clearly visible in the live tissue (Fig. S2). However, we obtained three grafts that clearly showed the injected dye in murine vessels after co-staining with CD31 and smooth muscle actin (Fig. 3A). While smooth muscle actin was detected around the arteries of the CAM vasculature as well as in the ureter of the grafts (Fig. 3A), no smooth muscle cells were detected around the CD31-positive murine endothelia, suggesting that the induction of flow was not sufficient to drive arterial differentiation and maturation (Fig. 3A,A′). We further noticed that all grafted kidneys displayed an unusually large density of capillaries in their outer regions compared to cultured kidneys and kidneys grown *in vivo* (Fig. S3). Taken together, these results indicate that the grafting of kidney explants results in neither an anatomically realistic nor in a mature vascular network.

To characterize the overall morphology of the grafts further, we stained three samples for the nephron progenitor marker Six2 and the collecting duct marker, E-cadherin (Fig. 3B). We noticed no gross abnormalities of the stained grafts compared to cultured kidneys (Fig. 3C). However, the grafting process did not appear to enhance growth of the kidneys compared to culture in type 1-collagen, in terms of branches and nephron progenitor clusters present. One of these explants further contained an unusual structure that emitted fluorescence in the channels used for Six2 and E-cadherin (Fig. 3B).

### Pre-existing arteries of E14.5 kidneys cannot be maintained by engraftment onto the CAM

**Fig. 4.**
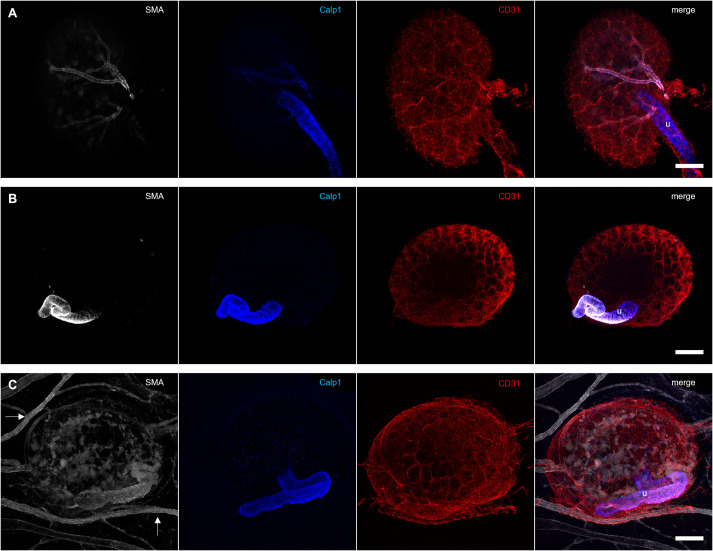
**Vascular smooth muscle cells are lost in grafted kidneys.** (A) Isolated E14.5 kidneys show strong smooth muscle actin (SMA) and weak Calponin 1 (Calp1) staining around the interlobular arteries. The ureter (u) stains strongly for Calponin 1. (B) E14.5 kidneys cultured for 3 days in type 1 collagen display a positive smooth muscle actin and Calponin 1 staining around the ureter, but none of the CD31-stained blood vessels are surrounded by smooth muscle cells. (C) Smooth muscle actin was detected in the galline vessels and around the ureter (u) of grafted E14.5 kidneys. Within the graft, patches with diffuse smooth muscle actin staining were visible. However, there was no alignment of smooth muscle cells around the murine CD31-positive capillaries visible. Calponin 1 was detected only in the ureter of the graft. Scale bars: 200 µm.

Our results showed that the flow provided by engraftment onto the CAM was not sufficient to induce smooth muscle cell recruitment. In order to determine whether it would suffice to maintain pre-existing arteries, we decided to engraft freshly isolated E14.5 kidneys, which already contain smooth muscle cell-lines interlobular arteries (Fig. 4A). When E14.5 kidneys were cultured within type 1 collagen, in the absence of flow, the arterial lining was lost (Fig. 4B), supporting the widespread hypothesis (Homan et al., 2019; Ryan et al., 2021) that blood flow is critical for arterial maintenance.

While some signal was detected from the smooth muscle actin antibody used to stain grafted E14.5 kidneys, the pattern did not reflect the arrangement of smooth muscle cells in freshly isolated E14.5 kidneys. The stain within the graft appeared more as randomly distributed clusters than as a coating of the renal arteries (Fig. 4C), suggesting that it originated from misplaced smooth muscle cells. The smooth muscle cell marker Calponin 1 was detected only around the ureter of the graft, not around any of the blood vessels. These results suggest that the flow provided by engraftment onto the CAM is not sufficient to maintain the arteries of E14.5 mouse kidneys. Interestingly, mature chick mesonephroi (temporary kidneys) grafted onto the CAM of quail eggs maintained their characteristic network of arteries and veins (Navarro et al., 2003). Organotypic kidney organoids grafted under the kidney capsule of adult mice display expression of the arterial marker Connexin40, indicating a higher degree of maturity compared to ungrafted organoids (Murakami et al., 2019). This suggests that species-specific differences in the haemodynamic profile or overall growth environment may prevent maturation and maintenance of the murine renal interlobular arteries on chick CAM. Using a murine host may therefore improve vascular maturity. However, Murakami et al. (2019) described that the vascular network detected in the organoids is different from that seen in kidneys grown *in vitro*. It remains to be identified to which extent this would impact the functionality of the organoids.

The data presented here suggest that the CAM assay is not suitable to achieve a mature and morphologically realistic vasculature in mouse kidneys. The grafted kidney explants contained mostly immature capillaries, which were present in an excess compared to what would normally be seen in kidneys grown *in vivo*. The vascular network further seemed to lack the hierarchical structure of arteries, capillaries and veins as there was no evidence of arterial smooth muscle cell recruitment. A prolonged *in ovo* incubation may improve vascular maturity; however, this would lead the other challenges such as the risk of immune rejection as the immune system of the chick becomes active around day 12 of incubation (Ribatti, 2016).

## MATERIALS AND METHODS

### Animals

Mouse embryos were obtained from pregnant CD-1 females, which were culled by a licenced staff member of the Bioresearch and Veterinary Service (University of Edinburgh) using a method listed under Schedule 1 of the UK Animals Scientific Procedures Act 1986. Fertilized Hy-line chick eggs were obtained from the National Avian Research Facility (University of Edinburgh). Care and use of experimental animals complied with the Animal (Scientific Procedures) Act 1986 and took place at an establishment licensed by the Home Office, according to Home Office and local guidelines and policies.

### Kidney culture and treatments

E11.5 kidneys were isolated and cultured in a 96-well plate on top of 50 µl type 1 collagen. After 2 days of culture in 100 µl KCM [MEM (Sigma-Aldrich) supplemented with 1.5% fetal bovine serum (Biosera) and 1× penicillin/streptomycin (Gibco)], explants were treated for 24 h with 100 µl KCM containing the indicated concentrations of the *Vegf* inducers progesterone (Sigma-Aldrich) and IGF1 (Biolegend). The pre-culture did not result in any reduction of CD31-positive structures between isolation and day 3, the time of grafting (Fig. S4).

### RNA isolation, reverse transcription and RT-PCR

E11.5 kidneys were pre-cultured for 2 days in KCM to increase the RNA yield. After treatment for 24 h with the indicated VEGF inducers following the pre-culture, RNA was isolated using an RNA Easy Mini extraction kit (Qiagen) according to the manufacturer's instructions. The cDNA synthesis was performed using 100 ng RNA in a volume of 14 µl. Following the addition of 1 µl (50 ng) prediluted oligo d(T) primer (Promega, C110A), the samples were heated to 70°C for 5 min and chilled on ice to break secondary structures. Subsequently, 10 µl master mix [0.625 µl RNAsin ribonuclease inhibitor (Promega, N251A), 5 µl M-MLV RT buffer (Promega, M531A), 1 µl M-MLV reverse transcriptase (Promega, M170A), 1.25 µl dNTPs (Promega, U144A) and 2.125 µl water] was added to each sample, and the samples were incubated for 1 h at 42°C. The reaction was terminated by heating the samples to 95°C for 5 min.

The obtained cDNA was diluted 1:4, and 2 µl of the diluted cDNA were used as a template for the RT-PCR reaction. The templates were combined with 5 µl 2× PowerUp Sybr Green Master Mix (Invitrogen) and 0.2 µl of each primer (Table S1). The Ct values of three technical replicates were averaged, and the relative expression to the two housekeeping genes (*Actb* and *Hsp90ab1*) was calculated using ΔΔCt method as described previously (Livak and Schmittgen, 2001).

### CAM grafting

Hy-line wild-type chicken eggs were maintained at 37.5°C and 60% humidity. After incubation overnight, 4 ml of albumin was extracted from each egg using a syringe with a 21G needle. After 6 days of culture, a window was cut in the shell above the CAM of the eggs and sealed with transparent tape. After an additional day of culture, the pre-cultured kidney explants were grafted onto the CAM. Prior to grafting, the CAM was gently abrased using a pipette tip. For initial experiments, which were aimed at optimizing the grafting efficiency, this was done using a wide orifice pipette tip (Starlab, E1011-8400) and moving it in a circular motion. During later experiments, which were primarily used to analyse the graft morphology, the injury was induced using a fine tip and moving it in a stroking motion across the grafting site. After 3 additional days of incubation, the CAM containing the grafted kidneys was extracted and fixed with PFA. To visualize the blood flow, the CAM vasculature was injected with 10 µl donkey-anti-mouse AlexaFluor594-conjugated antibody.

### Calculation of CIs of the difference of the proportion of grafted kidneys

To calculate the 95% CI for the difference between the proportion of grafted kidneys with preculture in grafting and control medium, the binomial normal approximation interval was used as described previously (Gardner and Altman, 1986).

### Immunofluorescence staining and clearing

Grafts that were injected with DAM555 were fixed with 4% PFA (in PBS, pH adjusted to 7.2 using NaOH) overnight at 4°C. Following fixation, the grafts were washed three times with PBS and permeabilized for 4 h in permeabilization buffer (20% DMSO, 300 mM glycine and 0.2% Triton-X-100 in PBS). Subsequently, the samples were incubated overnight in blocking buffer (3% donkey serum, 10% DMSO, 0.2% Triton-X-100 in PBS). The primary antibodies (Table S2) were diluted in primary buffer (5% DMSO, 3% donkey serum, 0.2% Tween-20) and incubated overnight at room temperature (RT). Subsequently, the samples were washed three times with PBS, and the secondary antibodies (Table S2) were added for overnight incubation at RT. After three final washes with PBS, the samples were dehydrated in graded alcohols (20%, 40%, 60%, 80%, 100% methanol) and transferred to 100% ethyl cinnamate (Merck) for clearing. The samples were cleared for a minimum of 24 h and imaged in ethyl cinnamate using an 18-well µSlide (ibidi).

Uninjected explants were fixed for 1 h with 4% PFA followed by dehydration in steps (20%, 40%, 60%, 80% and 100% methanol). Subsequently, autofluorescence was bleached by 1 h incubation in 5% hydrogen peroxide. Then, the kidneys were rehydrated in steps and washed three times with PBS. Subsequently, the bleached samples were incubated for 1 h in permeabilization buffer for 4 h at RT. Further staining and clearing was conducted as described above.

## Supplementary Material

Supplementary information
