# Supplementary material for: Introducing blood flow in kidney explants by engraftment onto the chick chorioallantoic membrane is not sufficient to induce arterial smooth muscle cell development
Source: Biol Open. 2022 Jul 6;11(7):bio059459. doi: 10.1242/bio.059459 (PMC9277080; doi:10.1242/bio.059459)
Supplement: Supplementary information [file biolopen-11-059459-s1.pdf]

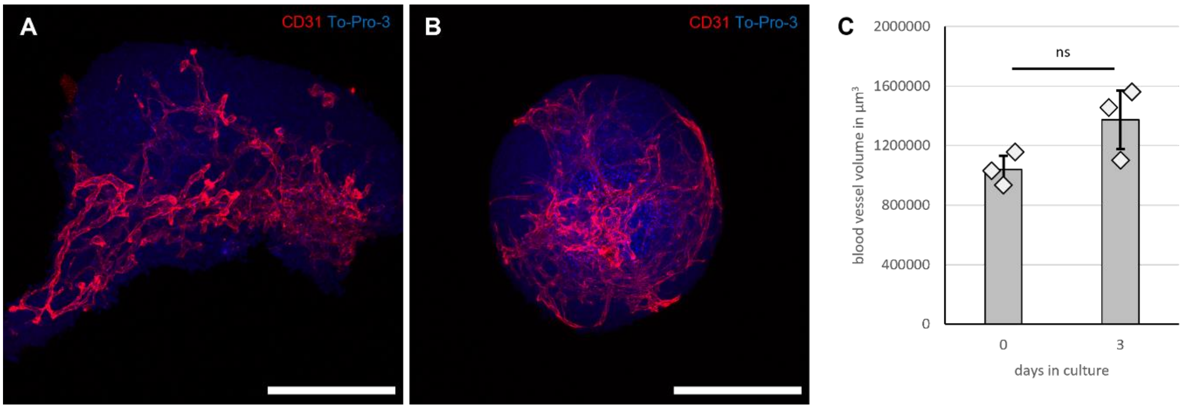

Fig. S1. Staining of freshly isolated (A) and 3-day cultured (B) E11.5 kidneys for CD31 (red) and ToPro 3 (blue). Scale bars: 200 µm. C: quantification of CD31 positive volume, difference not statistically significant according to Student's t-test.

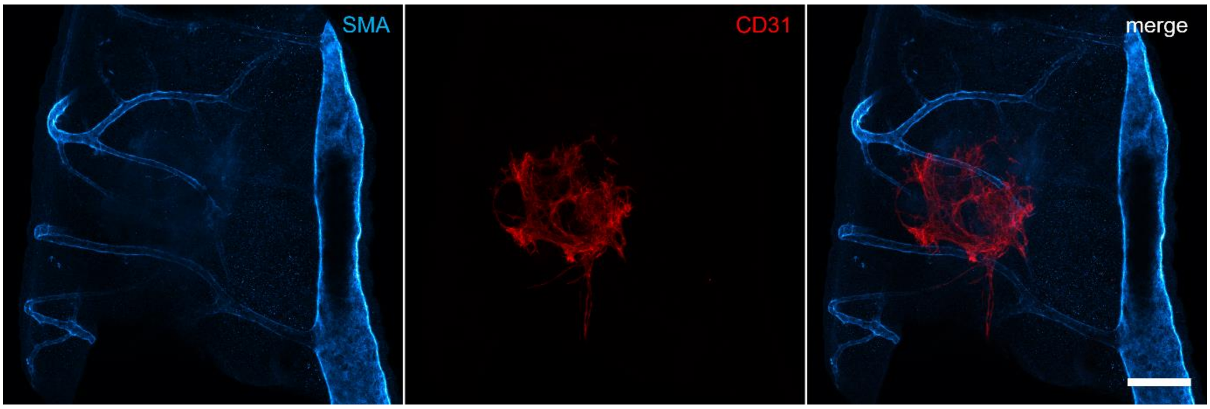

Fig. S2. Low magnification image of grafted kidney shows species specificity of CD31 antibody towards murine blood vessels. The vessels of the graft stained positive for CD31 (red), however the vessels of the surrounding CAM tissue which are visible though smooth muscle actin staining (cyan) display no CD31 stain. Scale bar: 200 µm

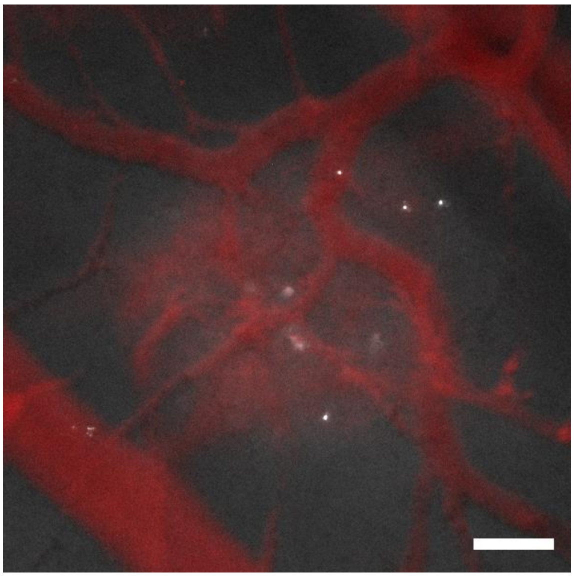

Fig. S3. Grafted kidney after injection of DAM555 antibody (red) shows perfusion of the graft. Scale bar 200 µm

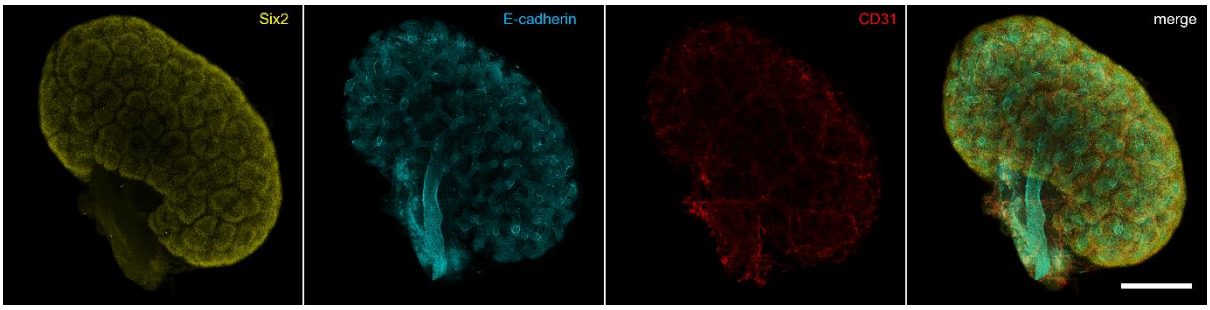

Fig. S4. Cortical capillaries of E14.5 mouse kidney. Z-projection of the cortical region of an E14.5 kidney stained with Six2, E-cadherin and CD31. The blood vessels grow around the cap mesenchyme but not through or cortical to it. Scale bar 200 µm

Table S1. Primer sequences for realtime PCR

Primer	sequence
**β-actin fwd**	GCCACCAGTTCGCCATGGAT
**β-actin rev**	GCCCACGATGGAGGGGAATACA
**Hsp90ab1 fwd**	CACCCTCTATTTGGAGAAGGAAC
**Hsp90ab1 rev**	CCATACTCCTCCTGCGTGAT
**panVEGFA fwd**	GCCAAGGCGCGCAAGAGA
**panVEGFA rev**	GCCTGGGACCACTTGGCA

Table S2. List of antibodies

Antibody	Supplier and catalogue number	Dilution
**Calponin 1**	Abcam ab46794	1:200
**CD31**	R&D AF3628	1:200
**E-cadherin**	BD 610182	1:200
**Podocalyxin**	R&D MAB1556	1:200
**Six2**	Proteintec 11562-1-AP	1:200
**Smooth muscle actin - FITC**	Merck F3777	1:100
**Donkey-anti-mouse AlexaFluor555**	Invitrogen A31570	NA
**Donkey-anti-mouse AlexaFluor488**	Invitrogen A21202	1:200
**Donkey-anti-rabbit AlexaFluor594**	Invitrogen A21207	1:200
**Chicken-anti-rat AlexaFluor594**	Invitrogen A21471	1:200
**Donkey-anti-goat AlexaFluor647**	Invitrogen A21447	1:200

Biology Open • Supplementary information

Table S3. Number of grafted kidneys for each condition and experimental run

experimental run	total number of kidneys in control group	number of successfully grafted kidneys from control group	total number of kidneys in P4/IGF-1 treatment group	total number of successfully grafted kidneys in P4/IGF-1 treatment group
**1**	10	3	6	0
**2**	8	2	4	2
**3**	14	2	16	0
**4**	10	2	8	1

Biology Open • Supplementary information

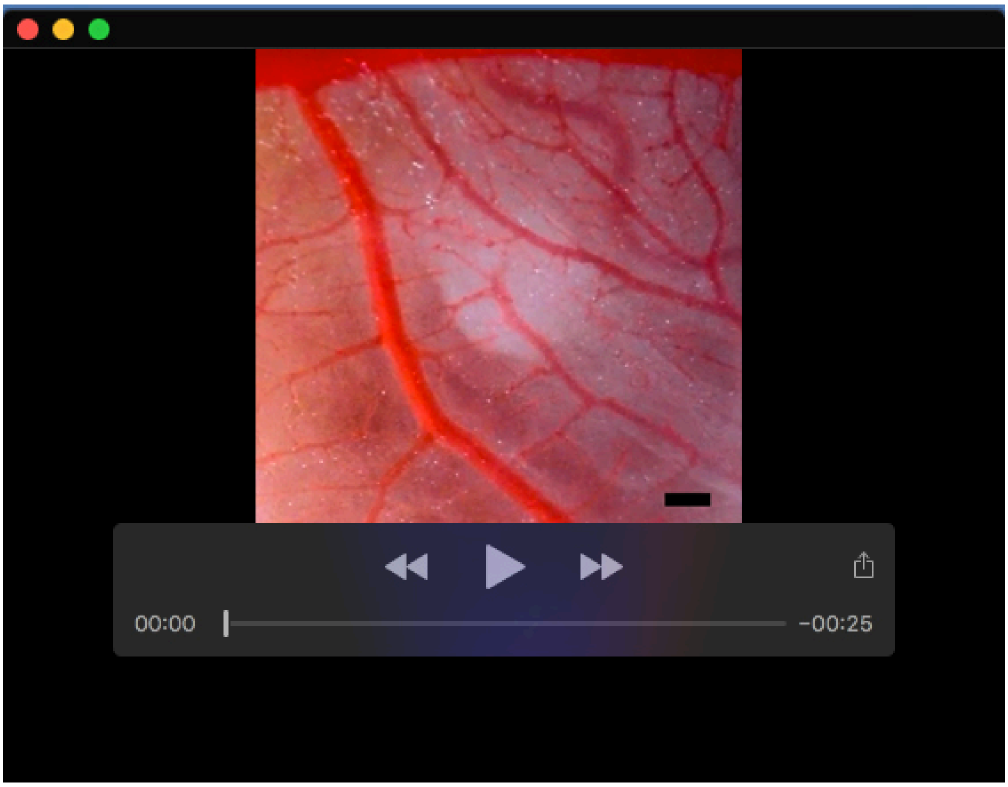

Movie 1. real-time imaging of graft using a digital microscope. Scale bar: 300 µm

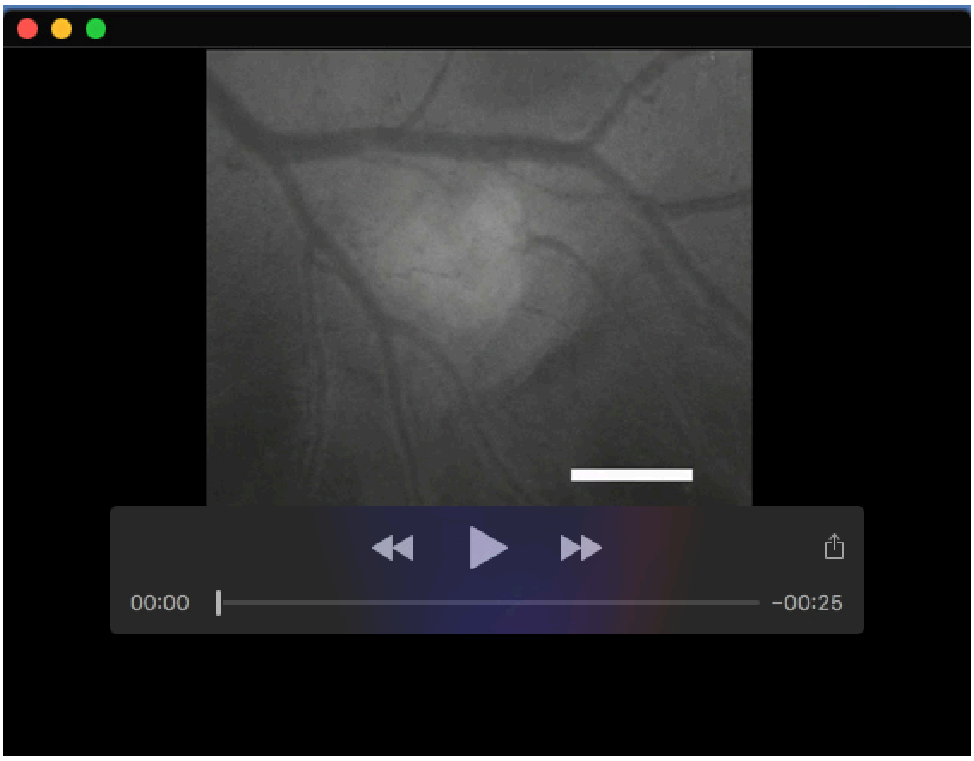

Movie 2. real-time imaging of graft after injection of Dextran-FITC. Scale bar: 300 µm
